# Gut Microbiome: A Potential Indicator for Differential Diagnosis of Major Depressive Disorder and General Anxiety Disorder

**DOI:** 10.3389/fpsyt.2021.651536

**Published:** 2021-09-13

**Authors:** Zaiquan Dong, Xiaoling Shen, Yanni Hao, Jin Li, Haoran Li, Haizheng Xu, Li Yin, Weihong Kuang

**Affiliations:** Mental Health Center of West China Hospital, Sichuan University, Chengdu, China

**Keywords:** gut microbiome, anxiety, depression, 16S ribosomal RNA, differential diagnosis

## Abstract

**Background:** Major depressive disorder (MDD) and general anxiety disorder (GAD) share many common features, leading to numerous challenges in their differential diagnosis. Given the importance of the microbiota–gut–brain axis, we investigated the differences in gut microbiota between representative cases of these two diseases and sought to develop a microbiome-based approach for their differential diagnosis.

**Methods:** We enrolled 23 patients with MDD, 21 with GAD, and 10 healthy subjects (healthy crowd, HC) in the present study. We used 16S rRNA gene-sequencing analysis to determine the microbial compositions of the gut microbiome based on Illumina Miseq and according to the standard protocol.

**Results:** GAD showed a significant difference in microbiota richness and diversity as compared with HC. Additionally, Otu24167, Otu19140, and Otu19751 were significantly decreased in MDD relative to HC, and Otu2581 and Otu10585 were significantly increased in GAD relative to MDD. At the genus level, the abundances of *Sutterella* and *Fusicatenibacter* were significantly lower in MDD relative to HC, and the abundances of *Fusicatenibacter* and *Christensenellaceae*_R7_group were significantly lower in GAD than in HC. The abundance of *Sutterella* was significantly higher whereas that of *Faecalibacterium* was significantly lower in GAD relative to MDD. Moreover, we observed that *Christensenellaceae*_R7_group negatively correlated with the factor score (Limited to Hopelessness) and total score of HAMD-24 (*p* < 0.05), whereas *Fusicatenibacter* negatively correlated with FT4 (*p* < 0.05). Furthermore, the GAD group showed significant differences at the genus level for *Faecalibacterium*, which negatively correlated with PTC (*p* < 0.05).

**Conclusions:** This study elucidated a unique gut-microbiome signature associated with MDD and GAD that could facilitate differential diagnosis and targeted therapy.

## Introduction

Major depressive disorder (MDD) is characterized by deep sadness, reduced energy, vegetative nervous system dysregulation, cognitive dysfunction, and even a high suicidal tendency (1). Generalized anxiety disorder (GAD) is characterized by extreme anxiety about issues, such as security, money, and health, and accompanied by restlessness and autonomic dysfunction (2). Anxiety and depression are two common disorders that show high comorbidity (3–5). Although they share several causal and descriptive features, there are some associated differences in their clinical features and etiological factors (6). The separation of anxiety and depression disorders is extremely important for the elucidation of the underlying disease mechanisms and development of specific pharmacological and psychological treatments. Although many studies have distinguished anxiety and depression from the perspective of symptomatology and psychological, social, and physiological etiology (7–10), there remains no convincing evidence of their distinction. To further elucidate the substantial but incomplete overlap between these disorders, this study sought to determine whether patients with clinical diagnoses of MDD and GAD can be differentiated based on gut-microbiota features.

The studies conducted in recent decades indicate that gut microbiota play a crucial role in modulating brain function and human behavior (11). Furthermore, differences in gut microbiota have been identified in various psychiatric diseases, including depression, bipolar disorder, and schizophrenia (12, 13), as well as several animal models of psychiatric diseases (14–16). There is evidence for altered microbiota composition in depressed individuals (17–19), with levels of *Faecalibacterium* negatively correlating with symptom severity (20) and suggesting that the clinical phenotype of mental illness might be affected by gut microbiota. Additionally, studies show that probiotic administration of *Bifidobacterium longum* and *Lactobacillus helveticus* can decrease anxiety (21–23). Moreover, Chen et al. and Jiang et al. found several consistent taxonomic differences, including higher abundances of Enterobacterales, *Bacteroidaceae, Escherichia/Shigella, Bacteroides*, and *Tyzerella* and lower abundances of Firmicutes, Mollicutes, *Prevotellaceae, Ruminococcaceae*, Subdoligranulum, *Coprococcus*, and *Dialister* between participants with GAD and controls (24, 25).

In general, recent studies independently investigated the characteristics of gut flora in depression and anxiety patients but did not conduct a comparative analysis. In fact, few studies have explored the use of gut flora as a marker for disease diagnosis. For example, recent studies suggest that changes in intestinal microflora might be used as a biomarker for depression diagnosis and monitoring (26, 27). Zheng et al. recently identified distinct gut-microbial compositions in MDD as compared with bipolar disorder and provided a novel marker panel to distinguish MDD from bipolar disorder based on gut-microbiome signatures (28). To the best of our knowledge, there is currently no information concerning differences in intestinal flora as a biological marker identifying anxiety and depression.

Emerging evidence points to a bidirectional communication between the neuroendocrine system and gut microbiota (29). Gut microbiota can modulate central processes via endocrine pathways within the microbiota–gut–brain axis (30, 31). Sudo et al. found that elevations in plasma adrenocorticotropic hormone (ACTH) and corticosterone levels in response to stress were substantially higher in germ-free mice than in specific pathogen-free mice (32). Additionally, recent studies report that germ-free mice, which are devoid of any bacterial contamination, show reduced depression-like behaviors along with changes in the hypothalamic–pituitary–adrenal (HPA) axis as compared with specific pathogen-free mice (33, 34). On the other hand, numerous studies have verified the relationship between the hypothalamic–pituitary–thyroid (HPT) or HPA systems and the onset of depression and anxiety (32, 35). For example, Brownlie et al. proposed that a dynamic decrease in thyroid hormone levels, particularly FT3 and FT4 (36), could be related to depression. Moreover, increased activation of the HPA axis has been repeatedly observed in depressed patients, especially in the melancholic subtype (37). In most studies, these two relationships [between depression/anxiety and gut microbiota and between neuroendocrine (HPA/HPT) and gut microbiota] have been studied independently. However, whether the gut microbiota can affect the neuroendocrine system and lead to mental illnesses, including anxiety and depression, remains unclear.

In this study, we compared the differences in the intestinal flora of patients with anxiety diagnosis to those with depression diagnosis in order to determine whether intestinal flora can help distinguish between the two groups. To achieve this, we examined MDD and GAD patients without obvious anxiety and/or depressive symptoms, respectively, and used 16S rRNA gene-sequence analysis to help distinguish differences in their intestinal flora. Additionally, we analyzed the effects of different bacteria on clinical symptoms and the neuroendocrine system in order to further explore their function in these conditions.

## Materials and Methods

### Subjects

Patients with MDD and GAD and normal control subjects participated in this study. Both MDD and GAD patients included a series of outpatients who received treatment at the West China Hospital from January to June 2019. All samples were from Chengdu, Sichuan, China, a relatively geographically closed area harboring residents with similar eating habits. The patients were diagnosed according to the Diagnostic and Statistical Manual of Mental Disorders (DSM-5) (38) at the first clinical examination, with diagnoses were confirmed by two psychiatrists (39). Patients <18 or >45 years of age and with organic etiology for their psychiatric symptoms, psychotic features, or intellectual disability were excluded. Patients included in the study were either newly diagnosed with depression or had not used psychotropic drugs for at least 6 months. The normal control subjects included 10 worker volunteers (aged 18–45 years) without current or past major psychiatric disorders. Subjects with the following conditions were also excluded: a lifetime history of bipolar disorder, schizophrenia, schizoaffective, or other psychiatric disorders; hypertension; cardiovascular disease; diabetes mellitus; obesity; liver cirrhosis; fatty liver disease; irritable bowel syndrome; inflammatory bowel disease; drug or alcohol abuse in the previous year; use of antibiotics, probiotics, prebiotics, or synbiotics in the 6 months before fecal sample collection; known active bacterial, fungal, or viral infections; and obvious dietary preferences (e.g., vegetarians). All patients completed the Hamilton Depression Rating Scale (HAMD-24) and the Hamilton Anxiety Scale (HAMA) to obtain a clinical rating of the severity of depression and anxiety (40, 41). Both scales were independently administered by two psychiatrists that were blinded to the clinical status of the participants and had attended a training session on how to administer the tests before the start of the study. To minimize the impact of accompanying symptoms, we also excluded GAD patients with HAMD-24 ≥20 and MDD patients with 14-item HAMA ≥14.

All procedures contributing to this work comply with the ethical standards of national and institutional committees on human experimentation and with the Helsinki Declaration of 1975, as revised in 2008. All procedures involving human subjects and patients were approved by the Ethics Committee of West China Hospital of Sichuan University (approval number: 2019-268). Written informed consent was obtained from all study subjects.

### Neuroendocrine Hormone Analysis

The HPT axis test indicators include thyroid-stimulating hormone (TSH; normal value: 0.27–4.2 mU/L), triiodothyronine (T3; normal value: 1.3–3.1 mmol/L), thyroxine (T4; normal values: 62.0–164.0 mmol/L), free triiodothyronine (FT3; normal value: 3.6–7.5 pmol/L), and free thyroxine (FT4; normal value: 12.0–22.0 pmol/L). The HPA axis test indicators include ACTH (normal value: 5.0–78.0 ng/L) and 8:00 A.M. cortisol (PTC; normal value: 147.3–609.3 mmol/L). Fasting venous blood was taken by drawing 4 mL of cubital venous blood at 8 A.M. after overnight fasting. All analyses were performed using a Roche Cobas e601 module (Roche, Basel, Switzerland) *via* electrochemiluminescence. All reagents and calibrations were performed according to manufacturer instructions.

### Sample Collection and DNA Extraction

Fecal samples were immediately frozen upon collection in a sterile plastic cup and stored at −80 °C before analysis. Microbial genomic DNA was extracted using the QIAamp DNA stool mini kit (Qiagen, Hilden, Germany) according to manufacturer instructions. The 16S rRNA V3-V4 amplicons were generated using the National Institutes of Health (NIH) Human Microbiome Project protocols (16S 454 Sequencing Protocol HMP Consortium; https://www.hmpdacc.org).

### 16S rRNA Gene-Sequencing Analysis

Libraries were prepared and paired-end sequenced with Illumina Miseq according to manufacturer instructions (42). These QIIME2 16S rRNA sequencing protocols were used to select and analyze operational taxonomic units (OTUs) (43). Sequences from this project were deposited in the NCBI Short Read Archive under BioProject ID PRJNA647236.

### Bioinformatic and Statistical Analyses

The sequence index file generated from the sequencing experiment was used to identify and extract the sample data saved in FASTQ format. Barcodes and the primers in the beginning and the end were used to identify and select sequence reads. The sequence number of each sample was normalized, and OTUs with 97% identity thresholds were used in the UPARSE (v.7.1; http://drive5.com/uparse/) software program. Chimeric sequences were identified and removed using UCHIME (v.4.1; http://drive5.com/uchime/). The taxonomy of each 16S rRNA gene sequence was analyzed with RDP Classifier (http://rdp.cme.msu.edu/) using the SILVA (SSU 138) 16S rRNA database at a confidence threshold of 70% (44).

Gut-microbiota-specific microbial characteristics were subjected to analysis of variance (ANOVA), emphasizing both statistical significance and biological relevance. ANOVA was used to compare the relative abundance of microbes identified with 16S rRNA sequencing.

Statistical analyses were performed using SPSS (v.21.0; IBM Corp., Armonk, NY, USA). One-way ANOVA was used to compare the continuous variables, including age, BMI, and clinical scales. Fisher's exact test was used to analyze contingency tables, and the chi-squared method was used to compare the variables of all three groups. A *p* < 0.05 was considered significant. The false recovery rate representing the threshold correction was generated using the Benjamini–Hochberg method (45).

The α-diversity was calculated by the ACE, Chao, Simpson, and Shannon indices. Mann–Whitney *U* tests were used to identify differences between the two groups. The β-diversity was calculated using the Bray–Curtis index as the distance method and reported according to principal component analysis. A hierarchical clustering tree was used to describe similarities among different data-point categories using the Bray–Curtis distance method and visualized using iTOL (https://itol.embl.de/).

PICRUSt, a bioinformatics software package that predicts metagenome functional content from marker gene (e.g., 16S rRNA) surveys and full genomes (46), was used to determine species function.

Circos software (http://circos.ca/) visualizes data in a circular layout (47) and was employed to visualize the relationship between samples and species.

Cytoscape is an open-source software platform for visualizing molecular-interaction networks and biological pathways and integrating these networks with annotations, gene-expression profiles, and other state data (48). We used this to determine the correlation between significantly different KEGG orthologs (KOs) and significantly different OTUs or species.

## Results

### Demographic Features and Levels of Neuroendocrine Hormone

We collected 54 fecal samples from the study participants, including 10, 23 (18 newly diagnosed and 5 relapsed), and 21 (19 newly diagnosed and two relapsed) subjects in the healthy crowd (HC), MDD, and GAD groups, respectively. The mean age at assessment was 30.04 ± 5.90, 30.43 ± 7.95, and 30.22 ± 6.50 years for the MDD, GAD and HC groups, respectively, with no significant difference found between groups according to ANOVA. Moreover, the BMI, sex ratio, marital status, family history, and levels of neuroendocrine hormones did not differ significantly among the three groups (Table 1).

**Table 1:** Clinical and demographic characteristics of the MDD, GAD, and HC groups.

**GROUP**	**MDD**	**GAD**	**HC**	***p***
Age (*y*)	30.04 ± 5.90	30.43 ± 7.95	30.22 ± 6.50	0.982
BMI	21.87 ± 3.00	21.19 ± 2.89	21.45 ± 2.80	0.743
HAMD-24	29.26 ± 7.51	12.10 ± 5.25	*NA*	<0.001
HAMA	8.00 ± 3.55	23.71 ± 7.30	*NA*	<0.001
Sex, *n* (%)				0.929^a^
Male	7 (30.43)	7 (33.33)	4 (40.00)	
Female	16 (69.57)	14 (66.67)	6 (60.00)	
Marital status, *n* (%)				0.935^a^
Never married	9 (39.13)	8 (38.10)	3 (30.00)	
Married	14 (60.87)	13 (61.90)	7 (70.00)	
Family history, *n* (%)			*NA*	0.481^b^
Yes	4 (17.39)	6 (28.57)		
No	19 (82.61)	15 (71.43)		
TSH	2.29 ± 1.32	2.55 ± 1.56	*NA*	0.543
TT3	1.50 ± 0.23	1.55 ± 0.30	*NA*	0.531
TT4	96.83 ± 14.95	88.22 ± 19.08	*NA*	0.102
FT3	4.54 ± 0.75	4.41 ± 0.77	*NA*	0.587
FT4	16.34 ± 2.71	15.04 ± 3.41	*NA*	0.168
ACTH	33.00 ± 17.77	29.83 ± 15.62	*NA*	0.535
PTC	389.58 ± 257.00	373.57 ± 267.43	*NA*	0.841

a *Fisher's exact probability method*.

b *Chi-squared test*.

### Diversity Analysis (α and β)

**Figure 1:**
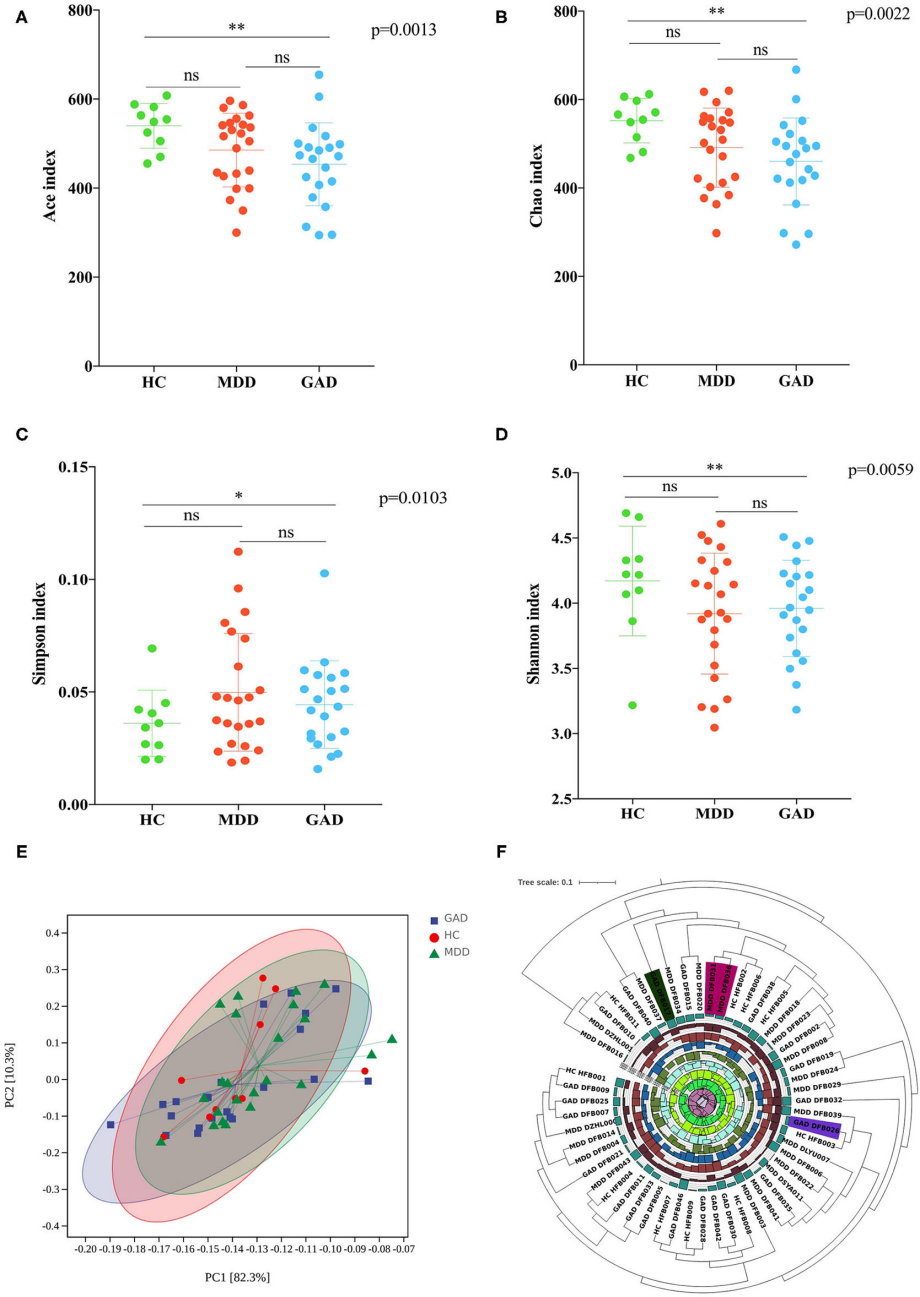
Diversity analysis (α and β). **(A–D)** Analysis of variations in richness (Chao and ACE indices) and diversity (Simpson and Shannon indices). MDD compared with HC revealed no significant difference in richness and diversity. GAD compared with HC revealed a significant difference in the richness and diversity. *p < 0.05 (Bonferroni < 0.017), **p < 0.01 (Bonferroni < 0.0033). MDD compared with GAD revealed no significant difference in richness and diversity. **(E)** Results of β-diversity visualized using principal component analysis (PCA; Bray–Curtis distance method). **(F)** Hierarchical clustering tree showing the similarities among different categories of data points by the Bray–Curtis distance method. Diversity analysis showed similar species diversity among the three groups.

Accounting for 70% of the valid sequences, we obtained 1,620,000 high-quality sequences from the 54 fecal samples of all participants: the HC, MDD, and GAD groups contained 630,000, 690,000, and 300,000 sequences, respectively. The richness of gut bacterial communities in all three groups was estimated by ACE and Chao indices, and the diversity was estimated using the Shannon and Simpson diversity indices. ACE and Chao analysis showed that most of the gut microbial diversity in each sample had been captured with the current sequencing depth. After rarefying the sequencing depth among all samples using a bootstrap method (30,000 reads per sample), the Shannon and Simpson diversity index estimates were calculated, revealing no significant difference in richness and diversity between HC and MDD. However, GAD showed a significant difference in microbiota richness and diversity as compared with HC (Figures 1A–D). To explore the differences in the comprehensive microbial phenotypes of MDD, GAD, and HC, we performed β-diversity analysis. Among the three groups, statistical analysis of β-diversity at the genus level indicated that the distance was similar according to principal component analysis (Figure 1E). The hierarchical clustering tree used to describe the similarities among different data point categories using the Bray–Curtis distance method showed sample similarities between the three groups (Figure 1F).

### Analysis of Fecal Species Community

**Figure 2:**
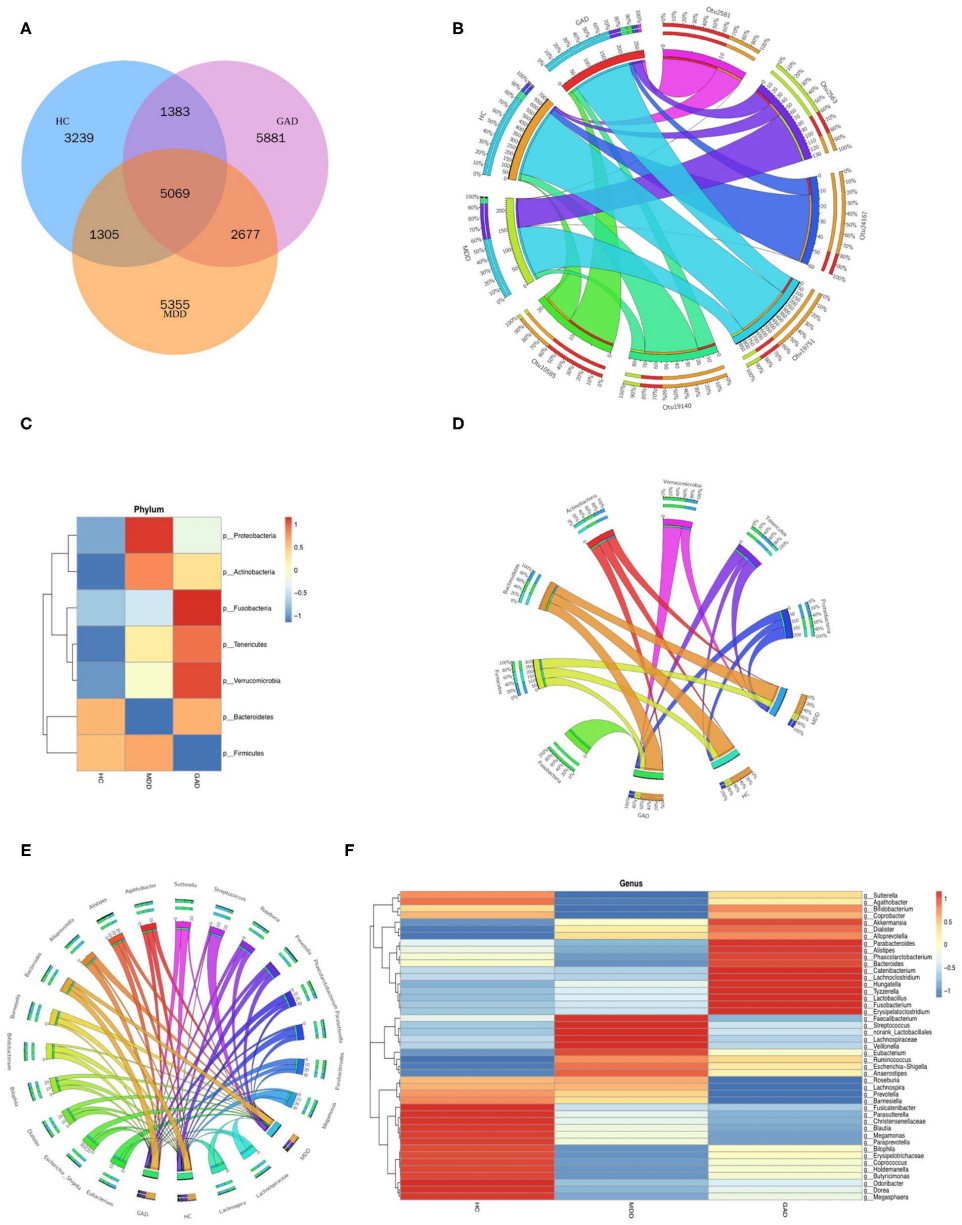
Species composition analysis. **(A)** Venn diagram of samples with common and unique OTUs. **(B)** Distribution of OTUs in the three groups. Data were visualized using Circos software (http://circos.ca/). The length of the bars for each sample on the outer ring represents the percentage of species in each sample. **(C,E)** Stacked bar plots at the phylum and genus levels show species composition according to the relative abundance of species. **(D,F)** Distribution of species in the three groups at the phylum and genus levels.

We obtained 10,996, 14,406, and 15,010 species-level OTUs from the HC, MDD, and GAD groups, respectively, using the OTU cluster method (Table 2). Results of the Venn diagram showed that 5,069 OTUs were common to all three groups (Figure 2A). The relationship between samples and OTUs is presented as the Circos diagram (Figure 2B). The general overview of gut bacterial composition at the phylum and genus levels is shown in Figures 2C,E. At the phylum level, the groups were rich in Bacteroidetes, Proteobacteria, and Firmicutes, but there were differences in terms of abundance. Compared with that in the HC group, we found that the relative abundance of Proteobacteria and Firmicutes increased considerably, and that of Bacteroidetes decreased considerably in the MDD group. Additionally, the abundance of Firmicutes decreased, whereas that of Bacteroidetes and Proteobacteria increased in GAD relative to HC vs. MDD (Figure 2C). The relationship between samples and phylum-level species is presented as the Circos diagram (Figure 2D). At the genus level, we found that *Bacteroides* and *Prevotella* were abundant in the three groups. The abundance of *Faecalibacterium* decreased and *Sutterella, Fusicatenibacter*, and *Christensenellaceae*_R7_group increased in MDD relative to HC. The abundance of *Fusicatenibacter* and *Christensenellaceae*_R7_group decreased in GAD relative to HC. Compared with that in the MDD group, we found an increase in the relative abundance of Fusobacteria, Tenericutes, Verrucomicrobia, and Bacteroidetes but a decrease in that of Proteobacteria, Actinobacteria, and Firmicutes in the GAD group (Figure 2E). The relationship between samples and species is illustrated by the Circos diagram (Figure 2F). In summary, we found that patients with MDD or GAD showed considerable changes in gut microbiota, and that there were differences in the relative abundance of gut microbiota in patients with both disorders.

**Table 2:** Comparison of phylotype coverage and diversity estimation of the 16S rRNA gene libraries at 97% similarity from the sequencing analysis.

**Group**	**No. of reads**	**No. of OTUs**	**Coverage (%)**	**Richness estimator**				**Diversity index**		
				**ACE**	**95% CI**	**Chao**	**95% CI**	**Shannon**	**Simpson**	**Evenness**
HC	630,000	10,996	97.19	4257.84	4031.74–4506.03	3090.89	2840.24–3395.07	4.733331714	0.032098	0.354556683
MDD	690,000	14,406	97.47	3762.74	3557.59–3989.13	2790.49	2562.74–3069.66	4.647938217	0.036133	0.34454694
GAD	300,000	15,010	96.15	5978.93	5689.91–6291.70	4170.2	3857.99–4538.97	5.017976	0.025108	0.397936241

*CI, confidence interval*.

### Analysis of the Signatures of Gut Microbiota

**Figure 3:**
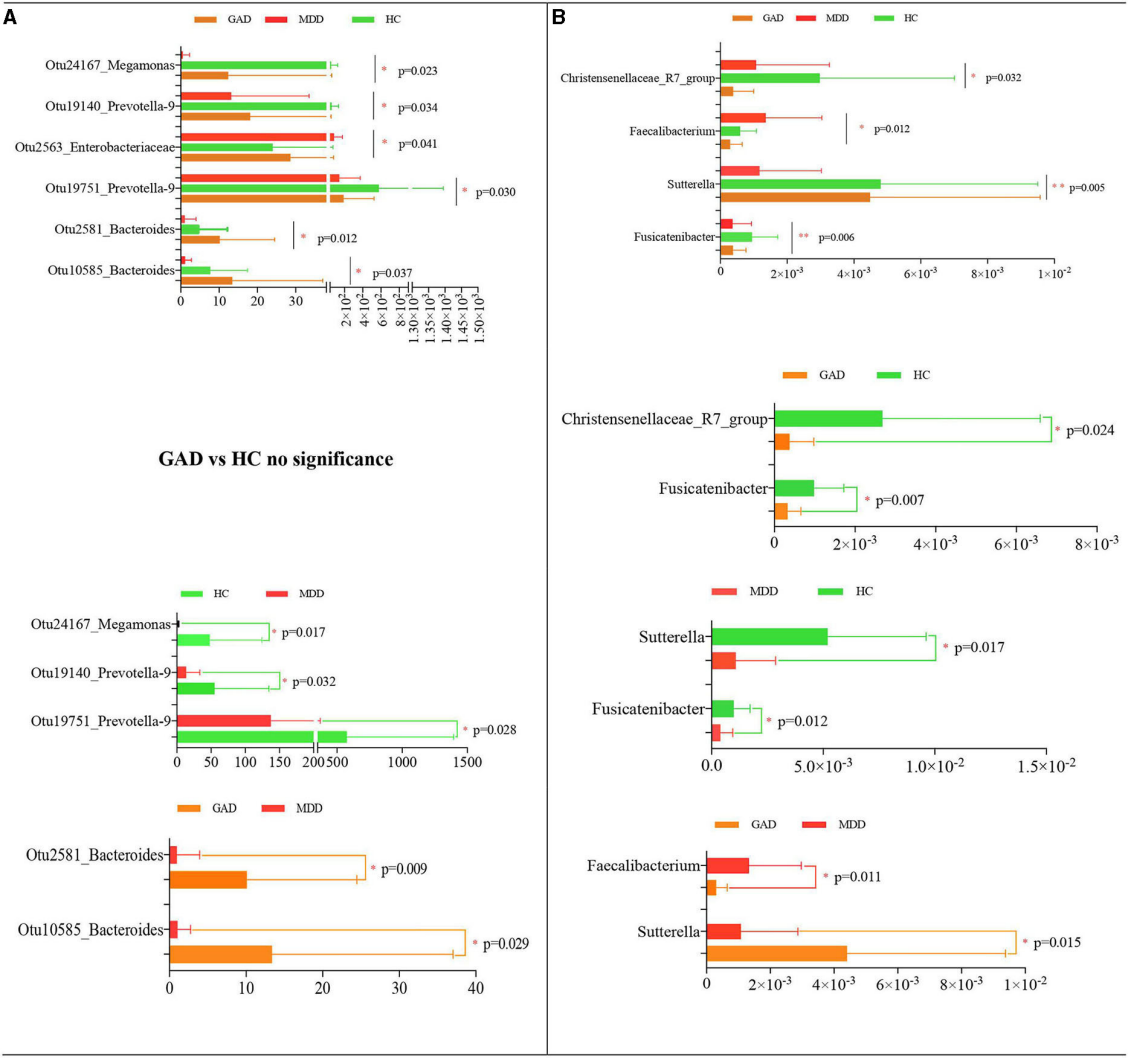
Statistical analysis of species differences. Taxonomic differences of fecal microbiota between HC, MDD, and GAD groups. Altered levels of specific bacterial OTUs and genera in MDD, GAD, and HC. **(A)** Six dominant bacterial OTUs were altered among the three groups. Otu24167, Otu19140, and Otu19751 were significantly decreased in MDD relative to HC. By contrast, Otu2563 was significantly upregulated in MDD relative to GAD or HC. Otu2581 and Otu0585 were significantly upregulated in GAD relative to MDD or HC. **(B)** Four significantly different species at the genus level across the three groups are shown. The abundances of *Sutterella* and *Fusicatenibacter* were significantly lower in MDD relative to HC. *Fusicatenibacter* and *Christensenellaceae*_R7_group abundances were significantly lower in GAD relative to HC. The abundance of *Sutterella* was higher, whereas that of *Faecalibacterium* was lower in GAD relative to MDD (all multiple comparisons; ANOVA tests).

We the compared the relative abundance of microbial composition among the three groups in the discovery set at both the OTU and genus levels. We found taxonomic differences in fecal microbiota among the HC, MDD, and GAD groups and identified six significantly different OTUs that were altered among the three groups (Table 3 and Figure 3A). The levels of Otu24167, Otu19140, and Otu19751 were significantly decreased in MDD relative to HC, whereas Otu2563 levels were significantly increased in MDD relative to GAD or HC. Otu2581 and Otu10585 levels were significantly increased in GAD relative to MDD. Furthermore, we found no significant difference in the abundance of OTUs between GAD and HC. Similarly, at the genus level, the abundances of *Sutterella* and *Fusicatenibacter* were significantly lower in MDD relative to HC, and both *Fusicatenibacter* and *Christensenellaceae*_R7_group abundances were significantly lower in GAD relative to HC. Additionally, the abundance of *Sutterella* was significantly higher, whereas that of *Faecalibacterium* was significantly lower in GAD relative to MDD (Table 4 and Figure 3B).

**Table 3:** Statistical analysis of the OTUs by ANOVA.

**Name**	**Mean (GAD)**	**Stderr (GAD)**	**Mean (HC)**	**Stderrn (HC)**	**Mean (MDD)**	**Stderr (MDD)**	***p***	**FDR**	***P* (HC-GAD)**	***P* (MDD-GAD)**	***P* (MDD-HC)**
Otu2581	10.0476191	3.1475434	4.5	2.42326685	0.91304348	0.63155822	0.01229678	0.8772808	0.3115826	0.00892584	0.60088641
Otu24167	12.2380952	10.253897	47.5	24.3284242	0.39130435	0.39130435	0.02301388	0.8772808	0.10009871	0.64423469	0.01727706
Otu19751	184.428571	73.8974377	572.4	260.248223	136.826087	49.1125453	0.03020169	0.8772808	0.06111859	0.92991901	0.02841122
Otu19140	18	7.43799768	54.5	25.2666887	13.0434783	4.26377251	0.03421628	0.8772808	0.07042385	0.91925868	0.03153303
Otu10585	13.3333333	5.16781861	7.6	3.10268701	1	0.36658881	0.03721526	0.8772808	0.60101603	0.0285381	0.50097406
Otu2563	28.5238095	10.8974919	23.9	15.2683041	79.4347826	20.067458	0.0418317	0.8772808	0.98528601	0.0650306	0.1230169

*FDR, false discovery rate; Stderr, standard error*.

**Table 4:** Statistical analysis of the species by ANOVA.

**Name**	**Mean (GAD)**	**Stderr (GAD)**	**Mean (HC)**	**Stderr (HC)**	**Mean (MDD)**	**Stderr (MDD)**	***p***	**FDR**	***P* (HC-GAD)**	***P* (MDD-GAD)**	***P* (MDD-HC)**
*Sutterella*	0.00439143	0.00108852	0.005187	0.00139934	0.00106652	0.00037587	0.00479284	0.66214192	0.85118087	0.01566996	0.01722476
*Fusicatenibacter*	0.00031762	7.44E-05	0.000974	0.00023634	0.00037304	0.00011939	0.00595727	0.66214192	0.00663858	0.93707457	0.01242988
*Faecalibacterium*	0.00029	7.86E-05	0.000552	0.00015174	0.00131739	0.00034548	0.01248295	0.70261169	0.81872373	0.01100806	0.1834709
*Christensenellaceae*_R7_group	0.00036429	0.0001336	0.002671	0.00124013	0.00101304	0.00045173	0.03155333	0.71208711	0.02401158	0.59744199	0.12743151

*FDR, false discovery rate; Stderr, standard error*.

### Functional Prediction of Gut Microbiota

**Figure 4:**
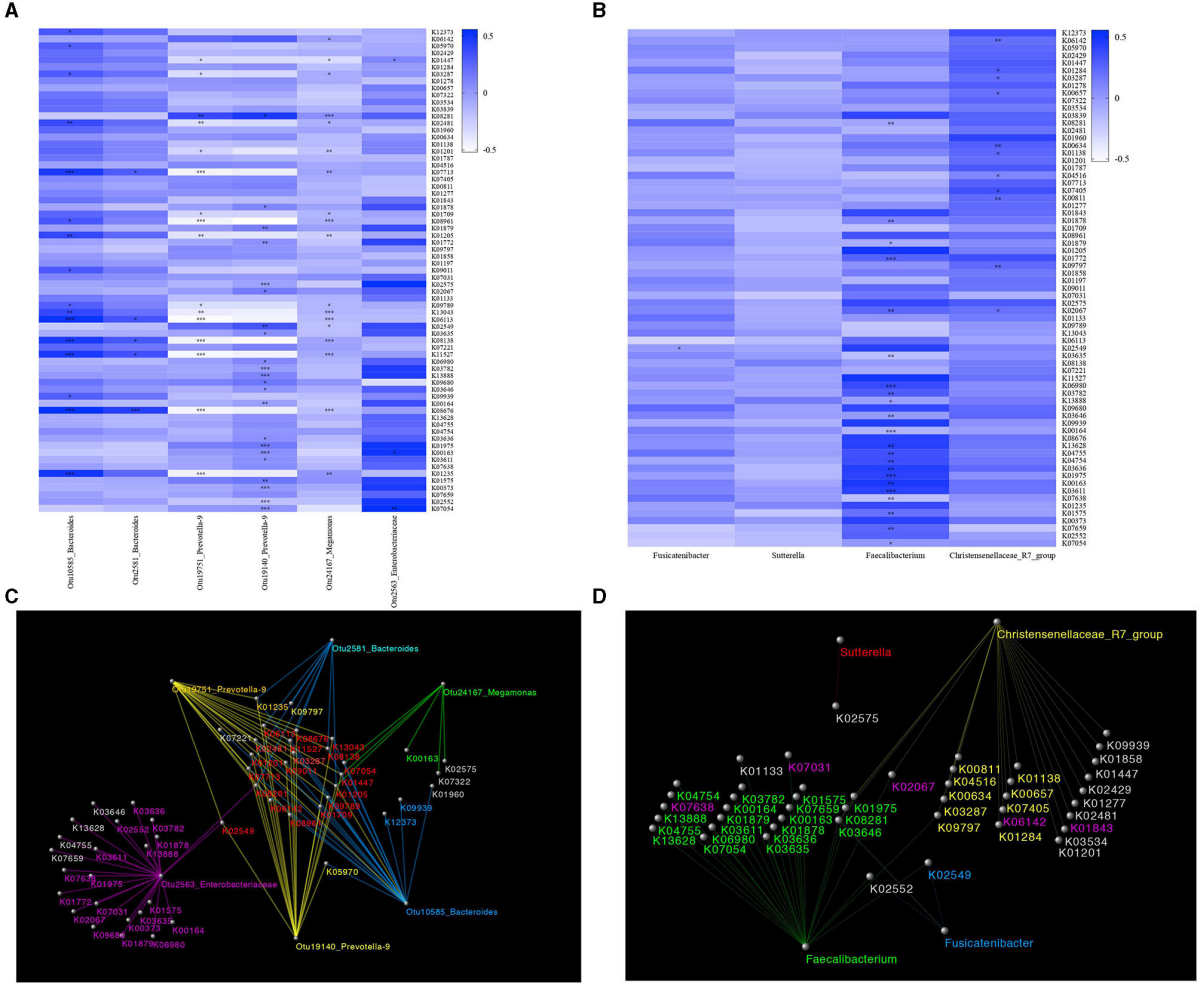
Functional prediction. Results of gut microbial functional pathway analyses. KOs were determined using PICRUSt. The significance of functional pathway prediction was determined by ANOVA (Supplementary Material). We obtained 69 significantly different KOs. The correlations between OTUs or species and KOs according to a heatmap are shown. Color variation indicates correlation, “+” indicates positive correlation, and “–” indicates negative correlation. *p < 0.05, **p < 0.01, ***p < 0.005. The network was used to show the correlation between significantly different KOs and OTUs or species using Cytoscape software. **(A)** The correlation between significantly different KOs and OTUs is shown in a heatmap. Sixteen KOs were significantly correlated with Otu10585_ Bacteroides, five KOs were significantly correlated with Otu2581_Bacteroides, 16 KOs were significantly correlated with Otu19751_Prevotella-9, 18 KOs were significantly correlated with Otu19140_Prevotella-9, 22 KOs were significantly correlated with Otu2563_Enterobacteriaceae, and three KOs were significantly correlated with Otu24167_Megamonas. **(B)** The correlation between significantly different KOs and species is shown in a heatmap. Eleven KOs were significantly correlated with *Christensenellaceae*_R7_group, 22 KOs were significantly correlated with *Faecalibacterium*, and one KO was significantly correlated with *Fusicatenibacter*. **(C)** Network diagram showing the relationship between significantly different KOs and OTUs. Different colors represent different OTUs. The same color between Kos and OTUs indicates a significant correlation. The red KOs represent significant correlations between KOs and multiple OTUs. **(D)** Network diagram showing the relationship between significantly different KOs and species. Different colors represent different species, and the same color indicates a significant correlation.

We obtained 6910 KOs and mapped them to the KEGG database using PICRUSt. A total of 69 significantly different KOs were obtained by ANOVA (Supplementary Material). The correlation between significantly different KOs and significantly different OTUs is depicted by a heatmap (Figure 4A). We found that the levels of two KOs (K01205 and K09011), which significantly correlated with Otu10585_Bacteroides, were significantly lower in MDD and higher in GAD relative to HC. The levels of one KO (K01201), which significantly correlated with Otu19751_Prevotella-9 and Otu19140_Prevotella-9, were lower in MDD and higher in GAD relative to HC. Another KO (K00163), which significantly correlated with Otu24167_Megamonas, showed higher levels in MDD relative to GAD and HC. Two KOs (K08281 and K03782), which significantly correlated with Otu2563_Enterobacteriaceae, showed higher levels in MDD relative to GAD and HC. Another KO (K07713), which significantly correlated with Otu2581_Bacteroides, showed lower levels in MDD relative to GAD.

The correlation between the significantly different KOs and species is depicted by a heatmap (Figure 4B). We found that two KOs (K00657 and K04516) significantly correlated with Christensenellaceae_R7_group, and that their levels were higher in GAD and lower in MDD relative to HC. Two KOs (K08281 and K02067) significantly correlated with *Faecalibacterium*, and their levels were higher in MDD and lower in GAD relative to HC. One KO (K02549) significantly correlated with *Fusicatenibacter*, and its levels were higher in MDD and lower in GAD relative to HC. We used a network diagram to explain the relationship between the significantly different KOs and OTUs/species (Figures 4C,D).

### Relationship Between Gut Microbiota and Clinical Parameters

**Figure 5:**
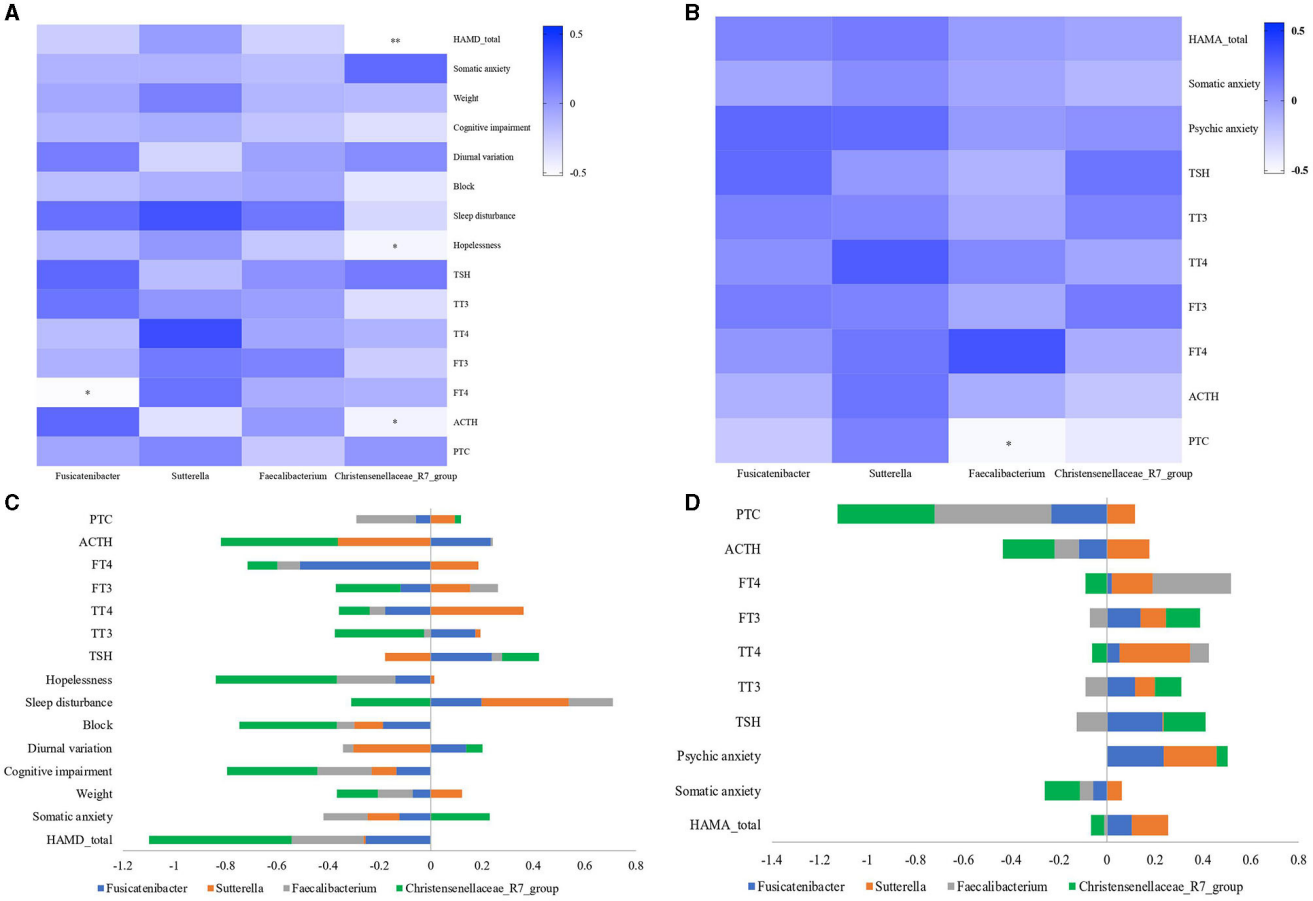
Relationship between gut microbiota and clinical parameters. Heatmaps were used to show relationships between significant differences in microbiota and clinical parameters, including hormones (PTC, ACTH, FT3, FT4, TT3, TT4, and TSH), the total and factor scores of HAMD (Hopelessness, Sleep disturbance, Block, Diurnal/variation, Cognitive impairment, Weight and Anxiety/somatic) in the MDD group, and the total and factor scores of HAMA (Psychic anxiety and Somatic anxiety) in the GAD group. **(A)** In the MDD group, *Fusicatenibacter* was significantly negatively correlated with FT4 (*p* < 0.05), and *Christensenellaceae*_R7_group was significantly negatively correlated with HAMD (*p* < 0.01), Hopelessness, and ACTH (*p* < 0.05). **(B)** In the GAD group, *Faecalibacterium* was significantly negatively correlated with PTC (*p* < 0.05). **(C,D)** The stacked bar plot was used to show the relationship between gut microbiota and clinical parameters (“–” shows a negative correlation, and “+” shows a positive correlation).

As noted, we found four bacterial genera (*Christensenellaceae*_R7_group, *Faecalibacterium, Fusicatenibacter*, and *Sutterella*) with differences in MDD and GAD relative to HC or between them. These four bacterial genera can be considered as important genera that affect the disease phenotype. To further explore the functions of these different bacterial genera, we evaluated correlations among the relative abundance of bacteria, hormones (including PTC, ACTH, FT3, FT4, TT3, TT4, and TSH), and the total and factor scores of HAMD (Hopelessness, Sleep disturbance, Block, Diurnal/variation, Cognitive impairment, Weight, and Anxiety/somatic) in the MDD group and those of HAMA (Psychic anxiety and Somatic anxiety) in the GAD group. We found significant differences in correlations at the genus level for *Fusicatenibacter* and *Christensenellaceae*_R7_group in MDD patients (Figure 5A). We observed that *Christensenellaceae*_R7_group negatively correlated with the HAMD factor score (Limited to Hopelessness) and total score (*p* < 0.05), *Fusicatenibacter* negatively correlated with FT4 (*p* < 0.05), and other key phylotypes showed no strong correlation. Further, the GAD group showed significant differences at the genus level for *Faecalibacterium*, which negatively correlated with PTC (*p* < 0.05), whereas other key phylotypes showed no strong correlation (Figure 5B). The columnar stack diagram shows the correlation between clinical parameters and the significantly different species (Figures 5C,D).

## Discussion

This study is the first to characterize and compare the gut-microbial compositions of patients with MDD and GAD. Our findings provide a better understanding of the differences between these two diseases in terms of their underlying mechanisms and will help in identifying novel therapeutic targets for better treatments.

We identified unique microbial signatures of patients with MDD and GAD relative to the HC. There was no significant difference in richness and diversity between the HC and patients with MDD. Consistent with this finding, numerous studies have reported no differences between MDD and control groups across all examined indices (49–51). However, some studies found decreased α-diversity in depressive disorders using the Shannon index (52, 53), as well as significant differences in β-diversity between participants with a depressive disorder and those in the control group (19, 52, 54–58). Based on the taxonomic findings in this study, Otu24167, Otu19140, and Otu19751 were significantly decreased in MDD as compared with HC, and the abundance of *Sutterella* and *Fusicatenibacter* at the genus level was significantly lower in patients with MDD than in the HC. Previous studies reported a lower *Sutterella* abundance in patients with depressive disorders than in the HC (19, 53–55); however, the association of *Fusicatenibacter* with depression has not previously been reported.

We found that the GAD group showed significantly higher microbiota richness and diversity than the HC, but there was no significant difference in OTU abundance between the two groups. This result differs significantly from previous reports. Chen et al. and Jiang et al. reported no difference in α-diversity between participants with an anxiety disorder and those in the control group, and that participants with GAD showed lower microbiota richness than control group subjects (24, 25). This heterogeneity of results might be attributed to numerous factors, including sample size, dietary intake, demographic characteristics of the participants, clinical status, sequencing methods, statistical methods, and/or the statistical significance threshold chosen to determine the disease-associated gut microbiota (59, 60). Based on the taxonomic findings (Figure 3B), *Fusicatenibacter* and *Christensenellaceae*_R7_group abundances were lower in the GAD group than in the HC. Mancabelli et al. reported *Christensenellaceae* as one of five taxa considered as a signature of a healthy gut (61). It is possible that its family might be related to affective disorders and neurological diseases (62). For example, patients with Parkinson's disease, multiple sclerosis, and autism have a remarkably lower relative abundance of *Christensenellaceae* (63–65).

The most important focus of this study was on distinguishing between MDD and GAD, and several significant differences were observed. Compared with patients with GAD, Otu2581 and Otu10585 levels were significantly reduced, whereas the abundance of *Sutterella* was decreased and that of *Faecalibacterium* was increased at the genus level in patients with MDD. However, no significant differences were observed in the α-diversity and richness of the intestinal floras between patients with GAD and patients with MDD, indicating that their intestinal floras were similar. Additionally, we found that Otu10585_Bacteroides significantly correlated with K09011 (map00290, valine, leucine, and isoleucine biosynthesis). Various studies also reported that MDD is associated with aberrant branched-chain amino acid and energy metabolism (66), suggesting that these amino acids (valine, leucine, and isoleucine) might serve as appropriate biomarkers for depression (67). We speculate that depression might be caused by the influence of Otu10585 on branched-chain amino acids metabolism. Moreover, the present results showed that *Faecalibacterium* was significantly correlated with K08281 (map00760, nicotinate, and nicotinamide metabolism) and K02067 (map02010, ABC transporters). Niacin deficiency is reportedly a contributing factor in mental-illness development and symptom alleviation (66). We speculate that the decreased abundance of *Faecalibacterium* might affect nicotinate and nicotinamide metabolism, leading to variations in correlative metabolism that result in MDD or GAD. ABC transporters exert notable effects on pathogen–host interactions and bacterial physiology (68), which might indicate another pathway of *Faecalibacterium* that results in GAD or MDD; however, the specific mechanism requires further study. Furthermore, the roles of *Sutterella* and Otu2581 remain unclear, although previous studies report that *Sutterella* is an intestinal flora associated with inflammatory responses and is found in abundance in autistic patients (69–71).

To determine why intestinal flora affect the clinical phenotype, we analyzed the correlation of some representative floras in patients with MDD or GAD with respect to the HPA or HPT axis. Our findings (Figure 5) implied that changes in intestinal flora might first induce changes in the HPA and/or HPT axis, which ultimately lead to the different clinical phenotypes of MDD and GAD. Previous reports indicated that gut-microbiota deficiency exacerbates the neuroendocrine and behavioral responses to acute stress (72–74). Other studies have also found a close relationship between HPT/HPA-axis dysfunction and depression/anxiety (32, 35, 36, 75, 76). For example, a dynamic decrease in thyroid hormone levels (particularly FT3 and FT4) is reportedly closely related to depression (36). These observations are consistent with the present findings and implications.

Additionally, we observed that *Christensenellaceae*_R7_group negatively correlated with factor (Limited to Hopelessness) and total scores of HAMD, suggesting that although *Christensenellaceae_R7_group* has not been observed as enriched in patients with MDD, it might affect the clinical manifestations and severity of MDD. Similar conclusions have been confirmed in other studies (20, 54).

This study has some limitations. First, the sample size was relatively small with no power calculation, which might have resulted in sampling bias. Second, the 16S rRNA gene sequencing used in this study resulted in limited functional information; therefore, whole-genome and whole-macrotranscriptome sequencing need to be performed in future studies. Third, we did not use standardized diagnostic tools to diagnose patients, assess the mental state of HCs and exclude comorbidities, which may weaken the reliability of the results. Last, other influential factors, such as food intake and physical activity, were not considered, which might also cause bias.

In summary, this study characterized and identified different gut-microbial compositions in subjects with MDD, subjects with GAD, and the HC. We identified a correlation between the bacteria and clinical symptoms, including a significant negative correlation between *Christensenellaceae_R7_group* and HAMD score. Moreover, we conducted a preliminary analysis of possible mechanisms underlying intestinal flora-affecting diseases. Our findings suggest that intestinal microflora might serve as molecular markers for distinguishing MDD from GAD.

## Data Availability Statement

The datasets presented in this study can be found in online repositories. The names of the repository/repositories and accession number(s) can be found in the article/Supplementary Material.

## Ethics Statement

The studies involving human participants were reviewed and approved by the Ethics Committee of West China Hospital of Sichuan University. The patients/participants provided their written informed consent to participate in this study.

## Author Contributions

ZD and WK: conception of the work, final approval of the version to be published, and agreement to be accountable for all aspects of the work. ZD, XS, YH, JL, HL, HX, and LY: acquisition of data. ZD and LY: analysis and interpretation of data for the work. ZD: writing. WK: revising the work. All authors contributed to the article and approved the submitted version.

## Funding

This work was supported by the National Natural Science Foundation of China [Grant Nos. 81621003, 81801350, and 81801357] and the Key R&D Projects of Science and Technology Department of Sichuan Province (Grant No. 2019YFS0217).

## Conflict of Interest

The authors declare that the research was conducted in the absence of any commercial or financial relationships that could be construed as a potential conflict of interest.

## Publisher's Note

All claims expressed in this article are solely those of the authors and do not necessarily represent those of their affiliated organizations, or those of the publisher, the editors and the reviewers. Any product that may be evaluated in this article, or claim that may be made by its manufacturer, is not guaranteed or endorsed by the publisher.
