# Supplementary figures and images for: Gut Microbiome: A Potential Indicator for Differential Diagnosis of Major Depressive Disorder and General Anxiety Disorder
Source: Front Psychiatry. 2021 Sep 13;12:651536. doi: 10.3389/fpsyt.2021.651536 (PMC8473618; doi:10.3389/fpsyt.2021.651536)

# ace curves

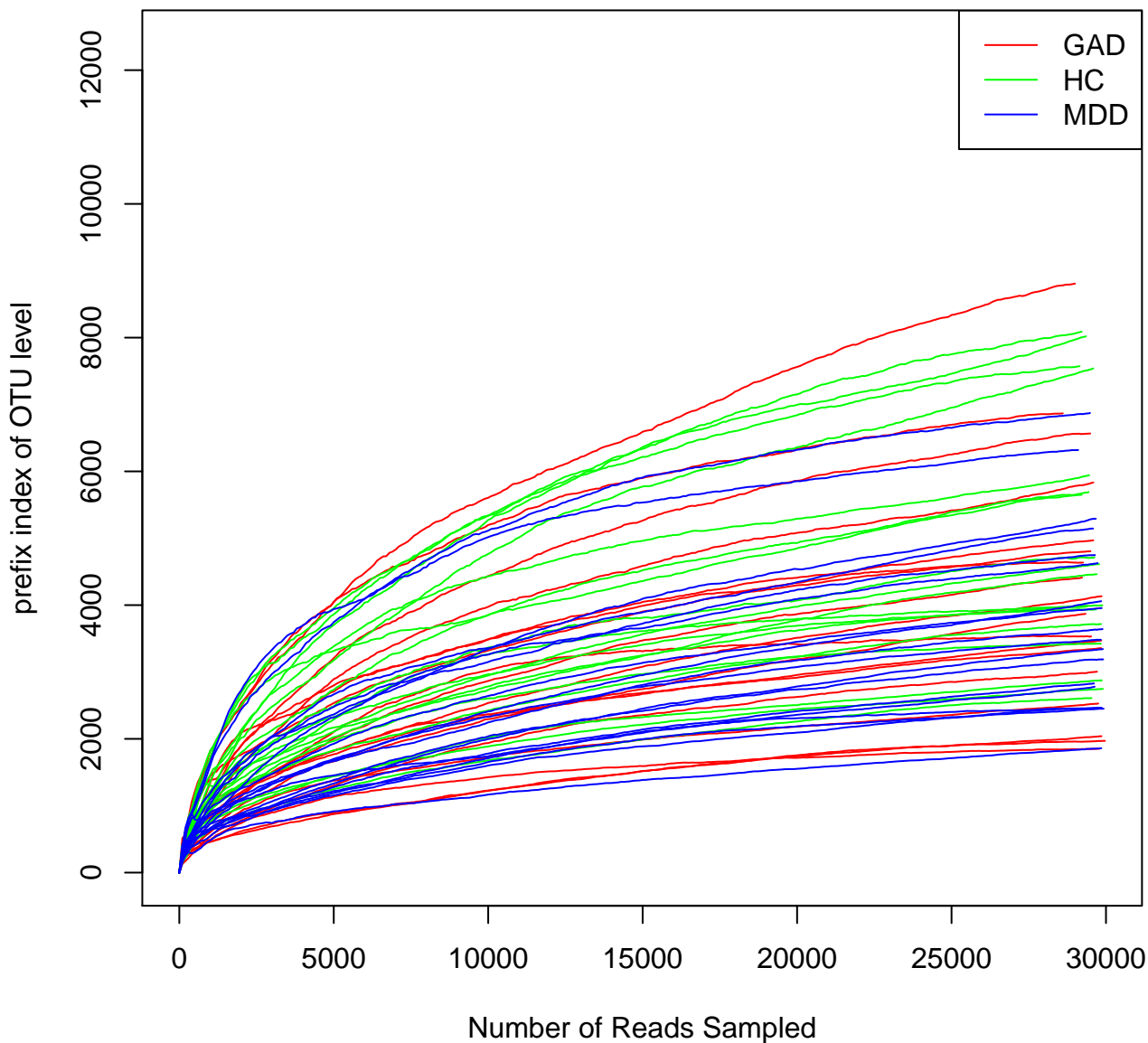

Supplement: Supplementary file 1 [file Data_Sheet_1.ZIP › Supplement/Rarefaction/ace.pdf]

# chao curves

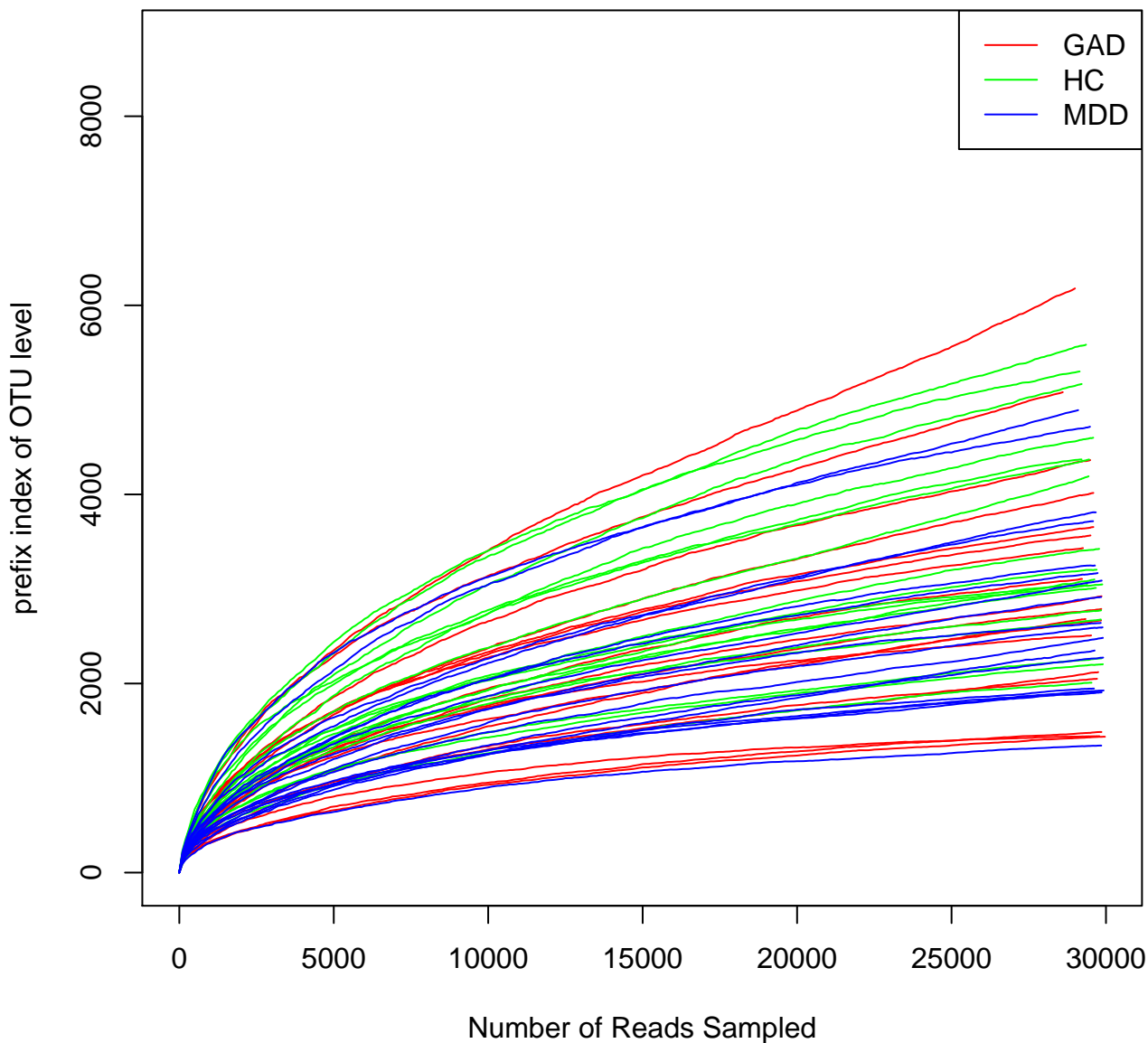

Supplement: Supplementary file 1 [file Data_Sheet_1.ZIP › Supplement/Rarefaction/chao.pdf]

**coverage curves**

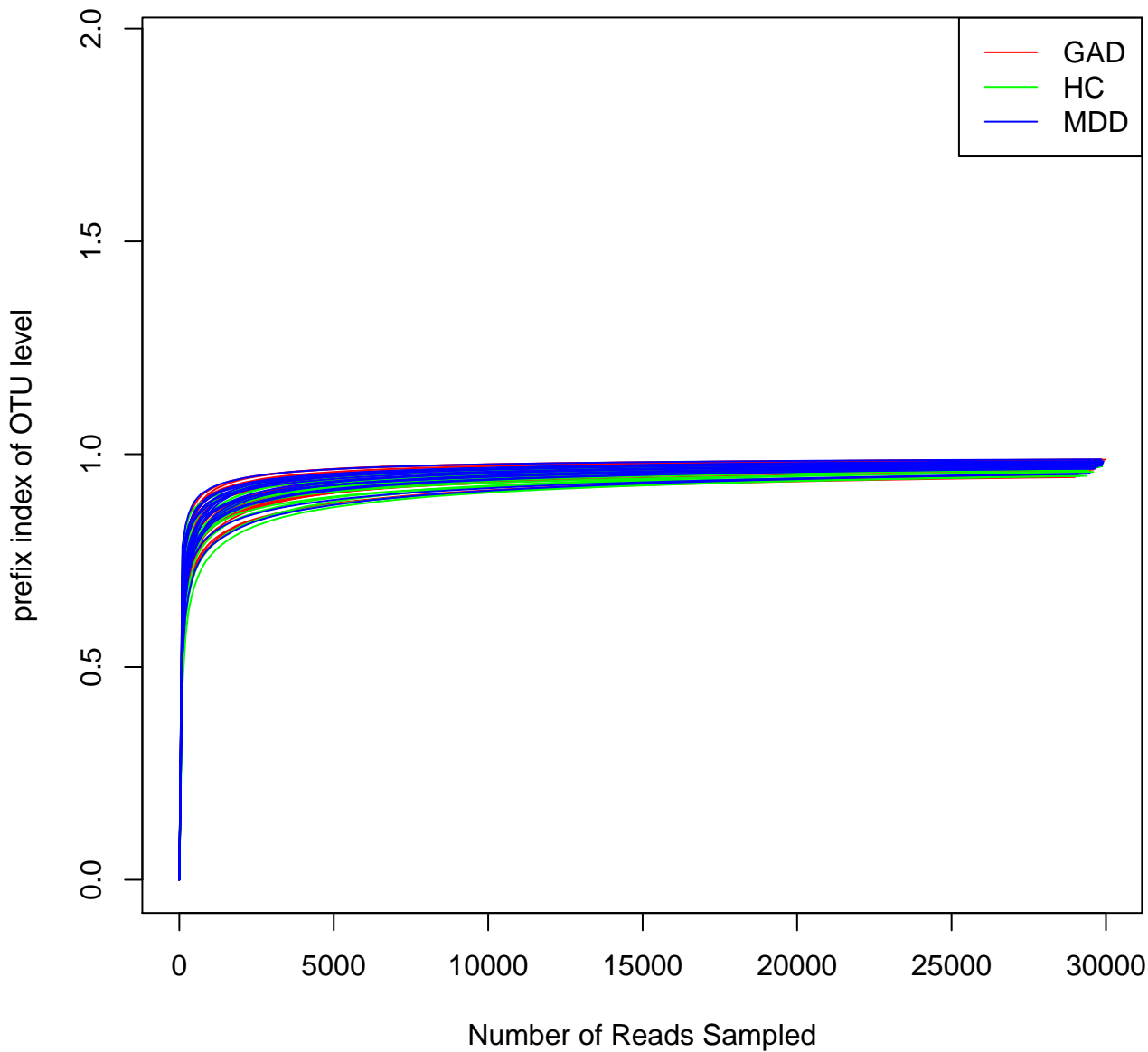

Supplement: Supplementary file 1 [file Data_Sheet_1.ZIP › Supplement/Rarefaction/coverage.pdf]

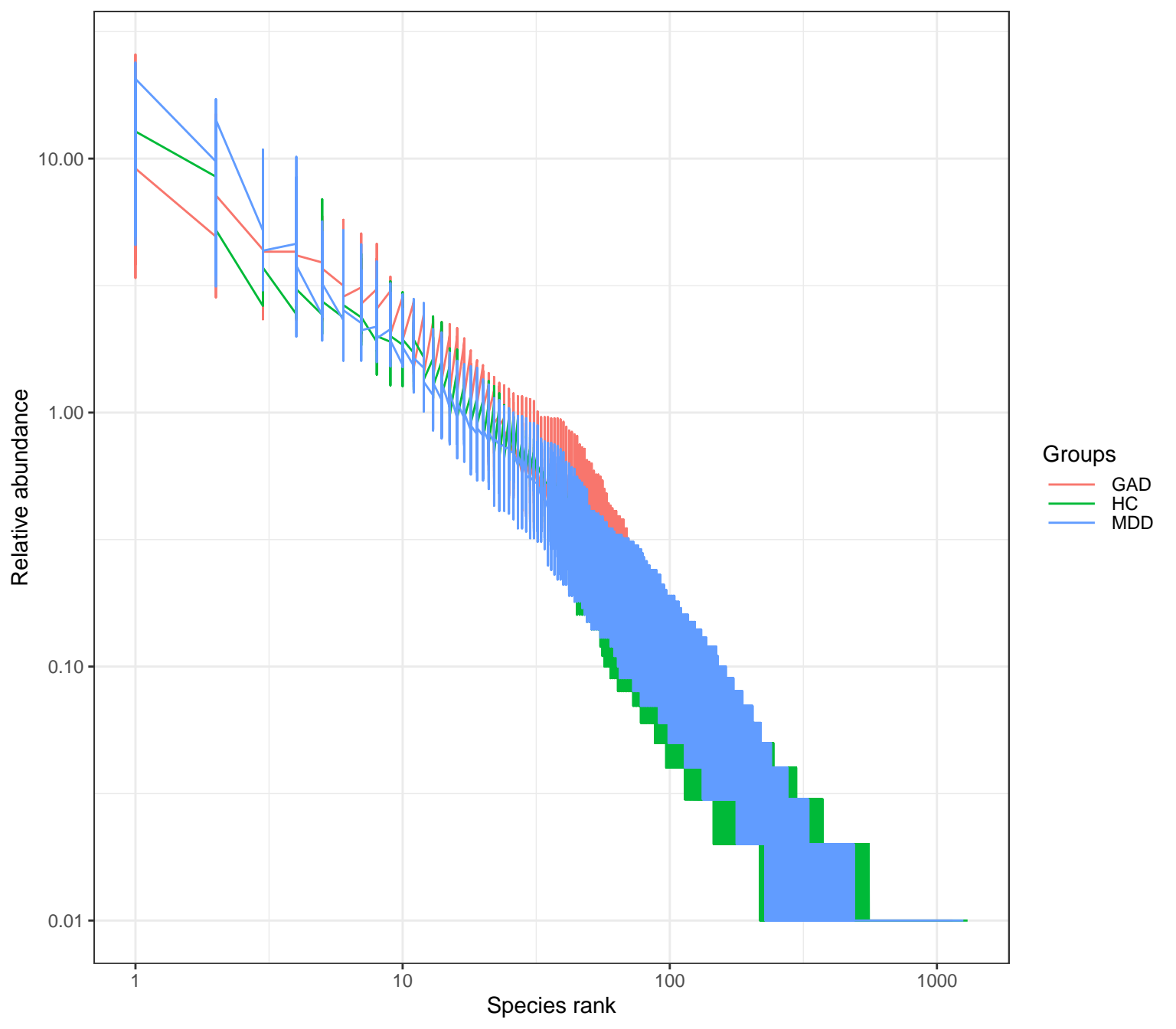

Supplement: Supplementary file 1 [file Data_Sheet_1.ZIP › Supplement/Rarefaction/rank_abundance.pdf]

# shannon curves

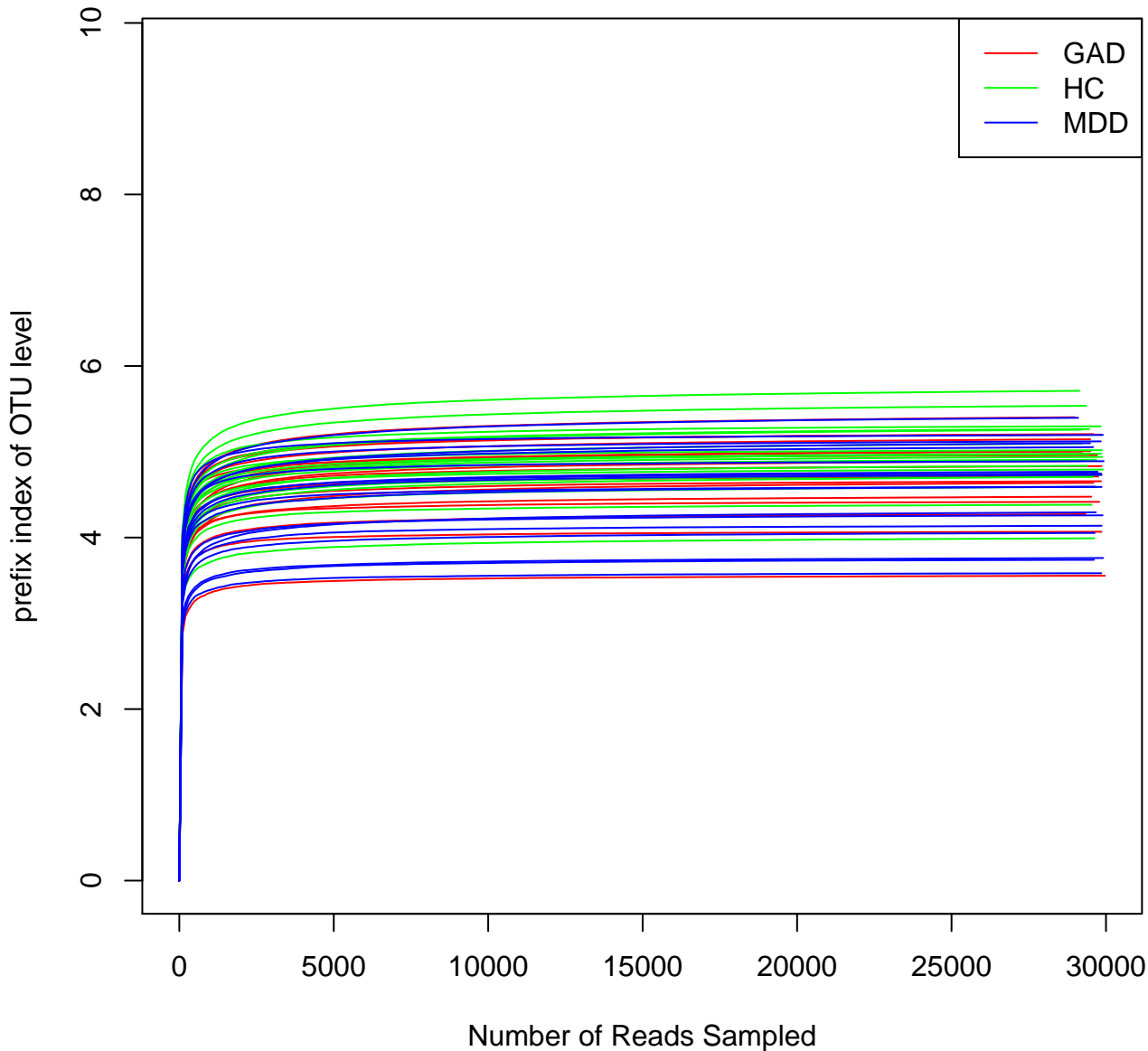

Supplement: Supplementary file 1 [file Data_Sheet_1.ZIP › Supplement/Rarefaction/shannon.pdf]

# simpson curves

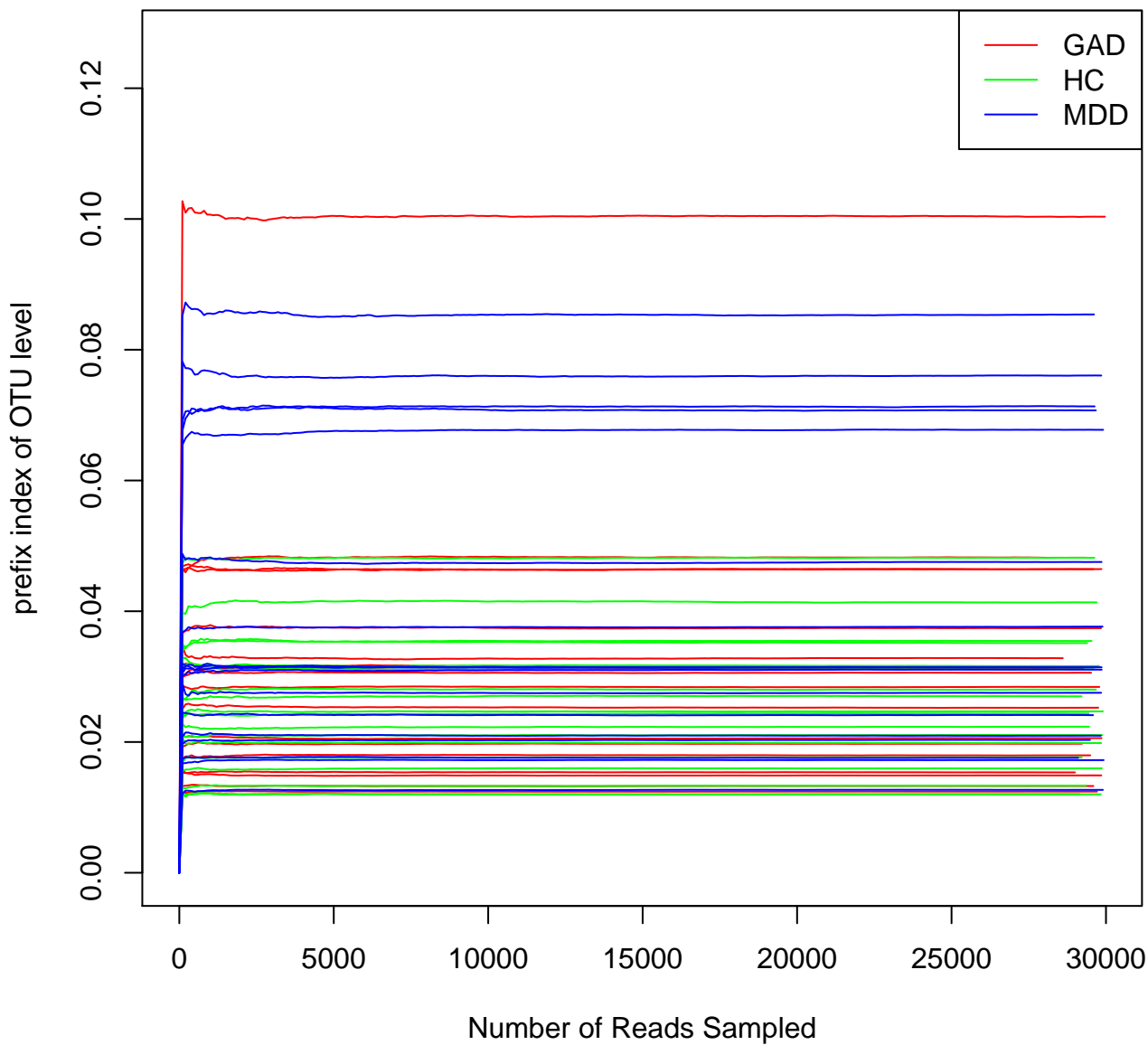

Supplement: Supplementary file 1 [file Data_Sheet_1.ZIP › Supplement/Rarefaction/simpson.pdf]

# sobs curves

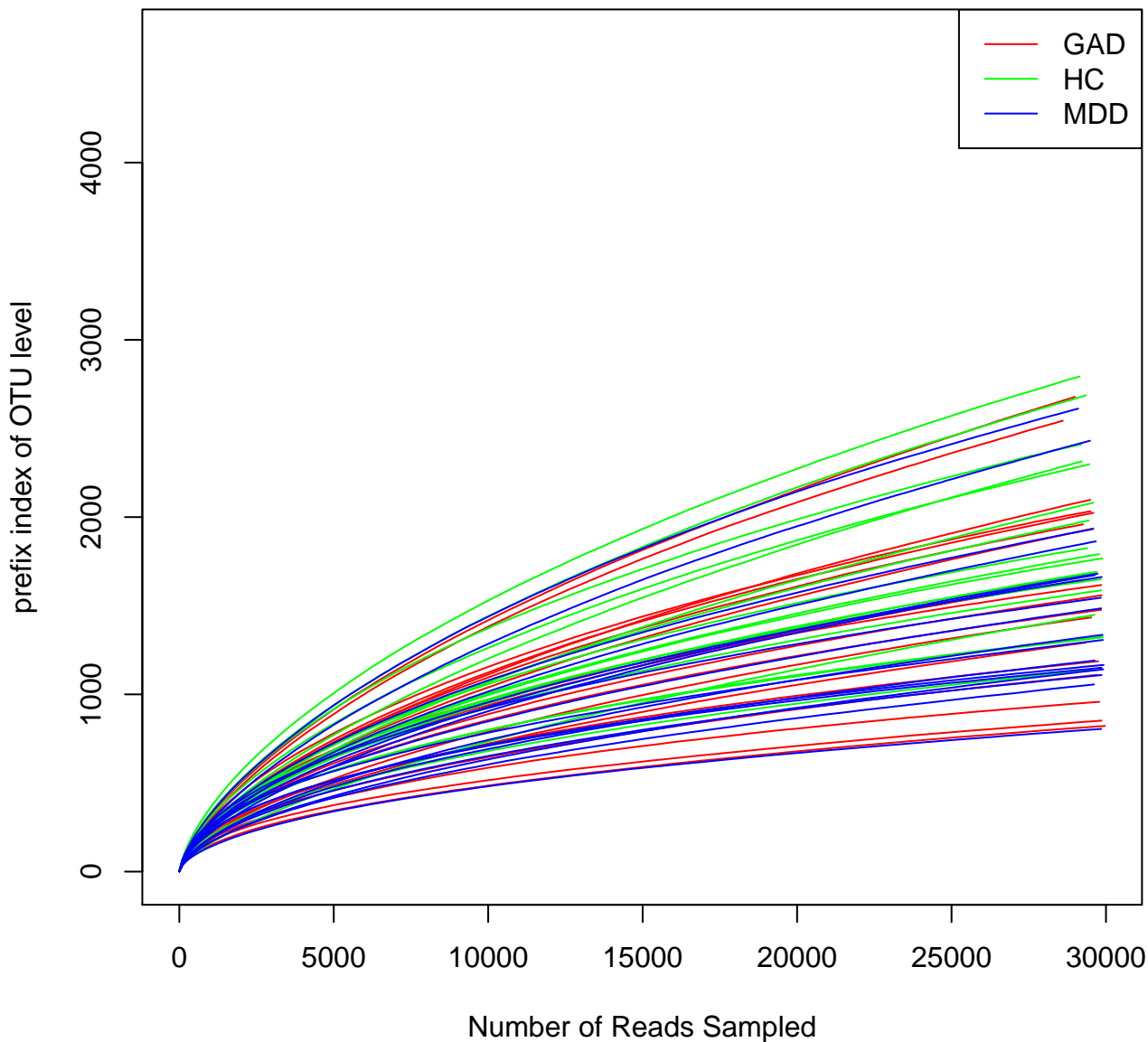

Supplement: Supplementary file 1 [file Data_Sheet_1.ZIP › Supplement/Rarefaction/sobs.pdf]
